# Dynamic Predictive Models With Visualized Machine Learning for Assessing Chondrosarcoma Overall Survival

**DOI:** 10.3389/fonc.2022.880305

**Published:** 2022-07-21

**Authors:** Wenle Li, Gui Wang, Rilige Wu, Shengtao Dong, Haosheng Wang, Chan Xu, Bing Wang, Wanying Li, Zhaohui Hu, Qi Chen, Chengliang Yin

**Affiliations:** ^1^ Department of Orthopedics, Xianyang Central Hospital, Xianyang, China; ^2^ Clinical Medical Research Center, Xianyang Central Hospital, Xianyang, China; ^3^ Department of Orthopaedics, Hainan Western Central Hospital, Danzhou, China; ^4^ Faculty of Science Beijing University of Posts and Telecommunications, Beijing, China; ^5^ Department of Spine Surgery, Second Affiliated Hospital of Dalian Medical University, Dalian, China; ^6^ Department of Orthopaedics, The Second Hospital of Jilin University, Changchun, China; ^7^ Department of Spine Surgery, Liuzhou People's Hospital, Liuzhou, China; ^8^ Microbial Resource and Big Data Center, Institute of Microbiology, Chinese Academy of Sciences, Beijing, China; ^9^ Faculty of Medicine, Macau University of Science and Technology, Macao SAR, China

**Keywords:** chondrosarcoma, multicenter, nomogram, web calculator, prediction model

## Abstract

Chondrosarcoma is a malignant bone tumor with a low incidence rate. Accurate risk evaluation is crucial for chondrosarcoma treatment. Due to the limited reliability of existing predictive models, we intended to develop a credible predictor for clinical chondrosarcoma based on the Surveillance, Epidemiology, and End Results data and four Chinese medical institutes. Three algorithms (Best Subset Regression, Univariate and Cox regression, and Least Absolute Shrinkage and Selector Operator) were used for the joint training. A nomogram predictor including eight variables—age, sex, grade, T, N, M, surgery, and chemotherapy—is constructed. The predictor provides good performance in discrimination and calibration, with area under the curve ≥0.8 in the receiver operating characteristic curves of both internal and external validations. The predictor especially had very good clinical utility in terms of net benefit to patients at the 3- and 5-year points in both North America and China. A convenient web calculator based on the prediction model is available at https://drwenle029.shinyapps.io/CHSSapp, which is free and open to all clinicians.

## Introduction

Chondrosarcoma is a primary malignant cartilage tumor (1) and becomes more common with increasing age (2). Surgical resection is the main standard management for chondrosarcoma. Several studies revealed that chondrosarcoma might be insensitive or even resistant to radiotherapy and chemotherapy (3–5). Due to its rare incidence among sarcomas (1), the management guidelines are inadequate to make informed patient treatment decisions. Therefore, an accurate evaluation of chondrosarcoma patient prognosis would help clinicians provide appropriate therapy.

Many factors influence survival in chondrosarcomas, such as age, presence of metastasis, site of metastasis, pathological stage of the tumor, and grade of tumor differentiation (6, 7). These variables were usually used as the single indicator to assess prognosis, which were inadequate to make accurate individualized survival predictions for chondrosarcomas (8–10). Clinical prediction models can inform patients and their physicians or other healthcare stakeholders about the likelihood of a patient developing a certain disease and help them make relevant decisions (11). Therefore, applying clinical prediction models to real-world problems can help detect or screen undiagnosed subjects who are at a high risk. In addition, clinical prediction models can predict the prognosis of individual patients, which has an important clinical value in today’s world of precision medicine.

Building clinical predictive models requires adequate data, but it is difficult for a disease such as chondrosarcoma, which has a low overall incidence in the population (12). The Surveillance, Epidemiology, and End Results (SEER) database has the most complete and comprehensive cancer incidence and survival registry in the United States (13). There are currently several studies of clinical prediction models for chondrosarcoma based on the SEER, including models for predicting lung metastases from chondrosarcoma (8, 14–16). However, these studies were all constrained in data sources from the SEER. There is no external data to enhance the clinical application of these models in different regions. Besides this, the limitations about the non-dynamic and complex way of using the nomograms may reduce the clinicians’ willingness to use them in the real world.

In this study, we used Least Absolute Shrinkage and Selector Operator (LASSO), Univariate and Cox regression (UCOX), and Best Subset Regression (BSR) to establish the risk features that affect the overall survival of chondrosarcomas. Furthermore, a prediction model for chondrosarcoma was constructed based on the SEER database and four Chinese medical institutes. A web tool involving the feasible model was developed for flexible visualization and clinical usage.

## Results

### Characteristics of the Training and Validation Cohorts

In total, 1,209 SEER chondrosarcomas and 104 Chinese multicenter chondrosarcomas are enrolled in this study, and the demographic, clinicopathological, and treatment characteristics are summarized in **Table 1**. Both cohorts were similar in survival time under 3 years and had the same onset location of chondrosarcoma. Compared with the SEER data, Chinese chondrosarcomas significantly had a lower mean age, a larger tumor size, and a higher proportion of metastases (including N, lung metastases, and bone metastases). Besides this, the Chinese cohort was mainly made up by the Han population, while the SEER data was major in Caucasians (85%), was minor in Black (8%), and had other ethnicities (with a tiny part of the Hans). The flow chart of the data collection and analysis is shown in **Supplementary Figure 1**.

**Table 1:** Baseline data table of the training group and the validation group.

Variable	Level	Multicenter data (*N* = 104)	Surveillance, epidemiology and end results data (*N* = 1,290)	*p*
Survival months, mean (SD)	NA	33.29 (24.03)	34.19 (24.16)	0.713
Age, mean (SD)	NA	49.61 (14.63)	53.44 (18.12)	0.036
Race (%)	Black	0 (0.0)	96 (7.4)	<0.001
	Other	104 (100.0)	77 (6.0)	
	White	0 (0.0)	1,117 (86.6)	
Sex (%)	Female	38 (36.5)	571 (44.3)	0.154
	Male	66 (63.5)	719 (55.7)	
Primary site (%)	Axis bone	58 (55.8)	677 (52.5)	0.39
	Bone of limb	38 (36.5)	544 (42.2)	
	other	8 (7.7)	69 (5.3)	
Laterality (%)	left	40 (38.5)	496 (38.4)	0.839
	Not a paired site	26 (25.0)	293 (22.7)	
	right	38 (36.5)	501 (38.8)	
T (%)	T1	47 (45.2)	716 (55.5)	0.022
	T2	38 (36.5)	389 (30.2)	
	T3	4 (3.8)	13 (1.0)	
	TX	15 (14.4)	172 (13.3)	
N (%)	N0	91 (87.5)	1,237 (95.9)	<0.001
	N1	9 (8.7)	11 (0.9)	
	NX	4 (3.8)	42 (3.3)	
M (%)	M0	93 (89.4)	1,215 (94.2)	0.084
	M1	11 (10.6)	75 (5.8)	
Radiation (%)	No	96 (92.3)	1,149 (89.1)	0.388
	Yes	8 (7.7)	141 (10.9)	
Chemotherapy (%)	No/Unknown	97 (93.3)	1,231 (95.4)	0.449
	Yes	7 (6.7)	59 (4.6)	
Bone metastases (%)	No	99 (95.2)	1,273 (98.7)	0.019
	Yes	5 (4.8)	17 (1.3)	
Lung metastases (%)	No	94 (90.4)	1,234 (95.7)	0.028
	Yes	10 (9.6)	56 (4.3)	
Surgery (%)	No	19 (18.3)	177 (13.7)	0.256
	Yes	85 (81.7)	1,113 (86.3)	
Lymph node dissection (%)	No	95 (91.3)	1,213 (94.0)	0.377
	Yes	9 (8.7)	77 (6.0)	

By chi-square test and t-test.

T, tumor volume; N, lymph nodes metastases; M, distant metastasis.

Furthermore, the correlation analysis showed significantly positive relations between M and lung metastases, race, and category. The relations between surgery and survival status, M, grade, and lung metastasis were significantly negative (**Supplementary Figure 2**).

### Establishment of Risk Factors

**Figure 1:**
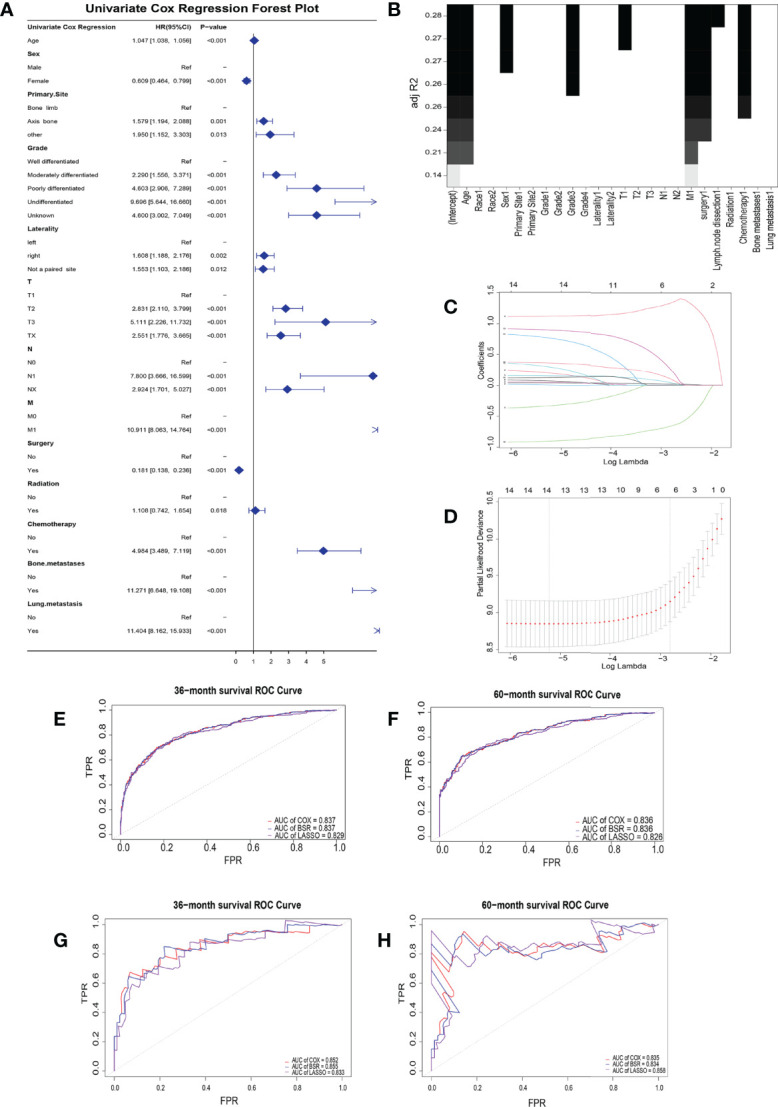
Clinical risk factor identification. **(A)** Forest plot about univariate cox regression. **(B)** Best subset regression. A graph was drawn with the adjustment *R*² as the criterion to see the combination of variables. **(C)** LASSO coefficient profiles of the 14 variables. When the *β* coefficient became zero, the variable made a negligible contribution to the model at this point and can be eliminated. **(D)** Partial-likelihood deviance curve for cross-validation of tuning parameter selection in the LASSO model. Two penalty values (tuning factors) *λ* were given: one is the value of *λ* when the mean squared error is smallest, *i*.*e*., *λ*.min; the other was the value of *λ* within a range of variance of *λ*.min. Receiver operating characteristic curves for the training **(E, F)** and validation **(G, H)** groups at 3- and 5-year overall survival.

Twelve indicative variables were screened by UCOX (*p* < 0.5): Age, Sex, Primary Site, Grade, Laterality, T, N, M, surgery, chemotherapy, bone metastases, and lung metastasis (**Figure 1A**). The BSR screened seven variables with max *R*²: age, sex, grade, T, M, surgery, and chemotherapy (**Figure 1B**). The LASSO regression analysis found the combination of six variables which have the best model performance and minimum variable number: age, grade, M, surgery, chemotherapy, and lung metastasis (**Figures 1C, D**).

The areas under the curve (AUCs) for the BSR, LASSO, and UCOX were all above 0.8 at different follow-up times (3 and 5 years), which confirmed that the three models had a good prognostic accuracy in the training cohort (**Figures 1E, F**) and the validation cohort (**Figures 1G, H**).

Furthermore, the combinations of variables from the above-mentioned three methods were included in the multivariate Cox analysis. Before the final models were determined, variable simplification was executed using stepwise backward regression with minimum Akaike information criterion (AIC) values. The AIC of the three models was 2,795.417 for the univariate Cox, 2,796.214 for the BSR, and 2,810.580 for the LASSO. As the UCOX has the smallest AIC value, the eight factors screened by stepwise backward regression were ultimately used to further construct the clinical prediction model.

### Risk Stratification of Key Factors

**Figure 2:**
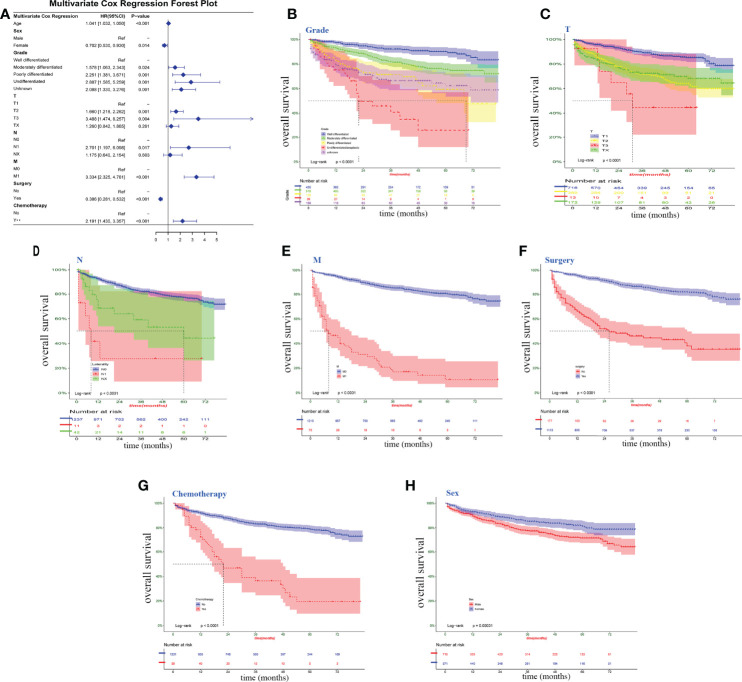
Clinical factors associated with survival risk. **(A)** multivariate Cox forest plot for the variables on Surveillance, Epidemiology and End Results data. **(B–H)** Kaplan–Meier survival curve; log-rank tests were performed for categorical variables. *P <*0.05, significant.

Eight clinical factors (age, sex, grade, T, N, M, surgery, and chemotherapy) from the univariate Cox regression showed a significant association with survival risk in the multivariate Cox forest plots and Kaplan–Meier survival curves (**Figure 2**). Six parameters were risk factors for overall survival in chondrosarcoma patients. The other two factors, being female and taking surgery, were the protective factors that could benefit the patients’ survival.

### Risk Predictor Construction and Validation

**Figure 3:**
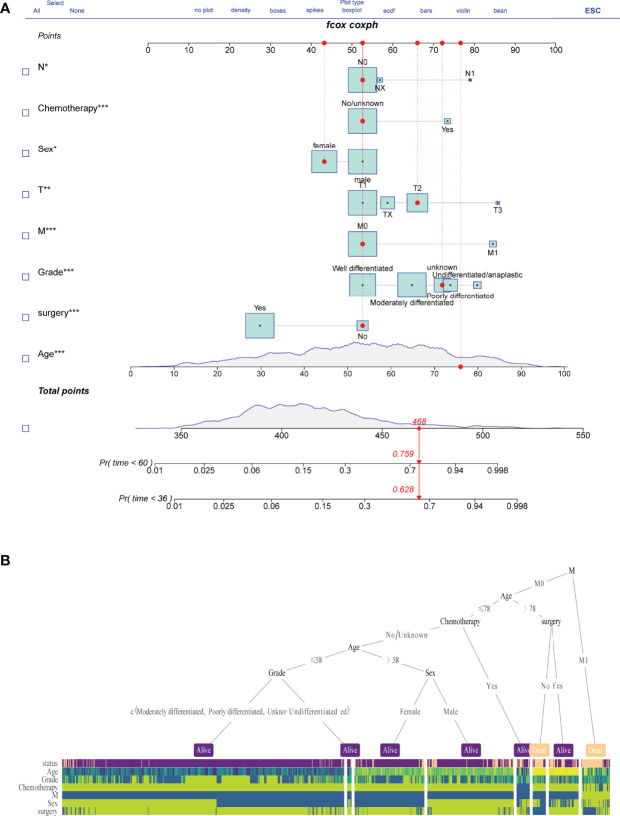
Nomogram model construction. Survival risk nomogram **(A)** and decision tree **(B)** for the prediction process.

A nomogram model for survival risk was constructed on the eight factors (**Figure 3A**), and a dynamic web calculator (https://drwenle029.shinyapps.io/CHSSapp/) based on the nomogram algorithm was designed to facilitate obtaining the chondrosarcoma survival probabilities. A decision tree based on key risk factors was developed to reveal mapping relationships between risk variables and predicted outcomes, which can potentially directly aid clinicians in the predicting process (**Figure 3B**).

**Figure 4:**
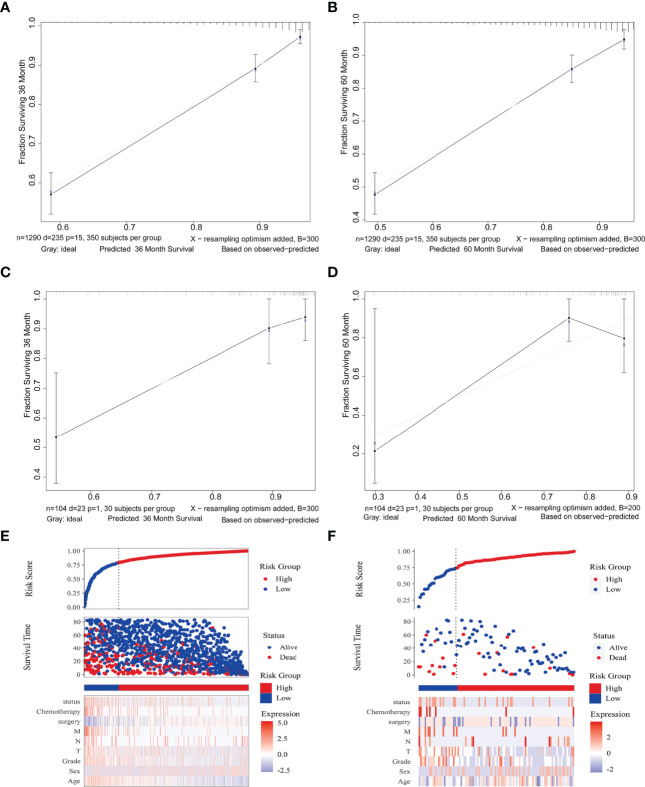
Nomogram model validation. Calibration diagram for internal **(A, B)** and external **(C, D)** cohorts. The x/y axes represent the predicted risk proportion to actual incidence, respectively. Risk factor association plots for the training **(E)** and validation **(F)** groups, respectively. Top, plots of risk scores; middle, scatter plots of survival time and survival status for high and low risk; bottom, heat maps of key value of risk factors.

To assess the prediction performance in reality, a calibration curve was plotted. For the different survival times of 3 and 5 years, both the SEER and Chinese multicenter cohorts revealed well compliance between prediction and actual incidence (**Figures 4A–D**). The risk score association further revealed the discrimination ability of the Cox survival risk models for both the training and the validation groups. Cutoff values were chosen to differentiate between patients with high and low risk, respectively, which have significantly different survival times and number of deaths (**Figures 4E, F**). These results suggested that the risk clinical signature provided additional value for personalized prognosis.

### Clinical Stratification With Predictor

**Figure 5:**
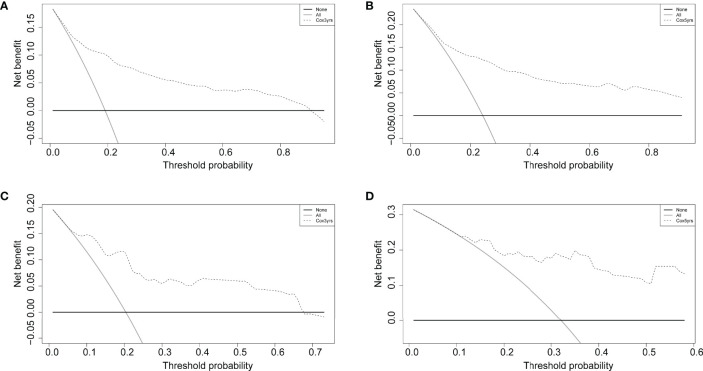
Decision curves of the nomogram comparison for the training **(A, B)** and validation **(C, D)** cohorts at 3 and 5 years. The solid black horizontal line represents no interventions triggered for all patients, the gray line represents all interventions triggered, and the dashed line is for the predictive model-guided trigger of medical interventions.

The decision curve analysis revealed that the clinical nomogram had a good clinical performance for both the training group (**Figure 5A, B**) and the validation group (**Figure 5C, D**). There were net benefits across almost the entire range of reasonable threshold probabilities in the USA and Chinese cohorts, which showed a general utility of survival probability prediction at both 3 and 5 years.

## Discussion

Chondrosarcoma is a rare primary malignant bone tumor with an incidence of about 1 in 200,000 (12, 17, 18). It is difficult to achieve practical prediction models due to the lack of clinical data. To our knowledge, this is the first study to use chondrosarcoma cohorts from different countries. Three different statistical methods were used simultaneously to screen the survival factors, and the clinical prediction models demonstrated an excellent discriminatory power and showed generalizability across countries and regions (which is integral to improving the prognosis).

For the first time, this study showed statistically significant differences between the chondrosarcomas of the USA and China at baseline, including clinical characteristics of age, race, tumor volume (T), and proportion of metastases (N, bone metastases, and lung metastases) (**Table 1**). The patients from the four regional medical centers in China were younger, with a mean age of 49.6 years, which was lower than that of the SEER database of 53.4 years. The SEER data categorized the people of Chinese descent as other, and the differences in ethnic composition of the two groups were even greater. However, the factor of race showed no significant survival risk in the methods of BSR, LASSO, and UCOX. The N1 patients with T2/3 showed higher proportions in lung and bone metastases, although there were no statistical differences when comparing the survival times and the Kaplan–Meier survival curves between the Chinese and the SEER data. These differences could be related to the more conservative health management philosophy in Chinese patients.

Currently, complete surgical resection with wide margins is the main treatment for chondrosarcomas, and the use of local adjuvant therapy remains controversial (19, 20). Studies showed that adjuvant chemotherapy after surgical resection has no significant benefit for most types of chondrosarcoma and even less so for grades II and III chondrosarcomas (3, 21). Our findings suggested that chemotherapy is even a risk factor for patient prognosis. Chondrosarcomas that are difficult to resect surgically due to tumor location, tumor size, or extensive metastatic disease have a 5-year survival rate of only 2% when chemotherapy has to be chosen as a treatment option (21). This may be the main reason why chemotherapy is a risk factor for prognosis. In this study, tumor metastasis (M) was negatively correlated with undergoing surgical resection and somewhat positively correlated with chemotherapy. The grade of differentiation was also negatively correlated with surgical resection. These reasons lead directly to the good correlation between primary tumor resection and survival. In previous studies, there were significant differences in the prognostic value of tumor differentiation grade on chondrosarcoma patient survival, which is also consistent with our study (17, 22, 23). This might be due to the heterogeneity of samples from conventional and non-conventional subtypes of chondrosarcoma (12). Among patients with chondrosarcoma presenting with metastases at diagnosis, those with resected primary tumors survived longer than those without (10). Therefore, every effort should be made to obtain surgical resection for patients presenting with metastatic chondrosarcoma when conditions permit.

Although the occurrence of lymphatic metastases in chondrosarcoma is very low, 0.9% in the SEER database, lymphatic metastases have a significant impact on patient prognosis and have been found to correlate strongly with pulmonary metastases (16). Clinicians should not neglect to examine the lymph nodes when making a diagnosis. Similarly, it was clearly observed in the nomogram that the higher the level of the T-stage, the worse the patient’s prognosis (**Figure 2C**). It was evidenced by recent studies reporting that tumor size is a significant independent predictor of mortality, which revealed that larger tumors are more proliferative and aggressive (24). Age and gender acted as independent risk factors affecting patient prognosis and were included in the prediction model. Unlike osteosarcoma and Ewing’s sarcoma, chondrosarcoma is usually seen in adults aged 40 to 75 years (1). The underlying disease and physical condition of older patients may not allow them to tolerate more radical treatment regimens, leading physicians to undertake more conservative treatment plans (9, 25). Besides this, several studies showed that being male is an independent poor factor for long-term prognosis in patients with chondrosarcoma (10, 24, 26). In a previous study of the risk of pulmonary metastases from chondrosarcoma, male patients have a higher risk of lung metastasis and a higher proportion of adverse habits such as smoking and alcohol abuse (16).

To our knowledge, although several web calculators have been studied in oncological diseases (27–29). This study is the first to use a web calculator based on a nomogram for chondrosarcoma. Compared to the previous traditional nomogram for chondrosarcoma, the web calculator can be used at a lower barrier and cost (8, 14–16, 30). It is easy to use, even for patients with no medical background or family members, and requires only a smartphone with an Internet connection. The patients can be conveniently informed about the progress of their disease and the possible future prognosis. The use of the web calculator also provides a tool for remote medical assessment, allowing doctors and patients to assess a patient’s condition more accurately without direct contact and providing an objective basis for guiding further medical decisions. The value of these clinical uses is even more pronounced in the context of the global outbreak of the new crown epidemic (31).

It is important to note that this is a retrospective study and there is a possibility of bias in the collection of multicenter data, which has a higher proportion of more severe diseases in the validation set. Future updates and prospective studies of the model are still needed to improve its accuracy. Factors such as surgical margins and specific chemotherapy regimens, which may affect patient prognosis, are lacking in the SEER database. Future data about chondrosarcoma patients treated with surgery should be collected to develop predictive models that affect surgical outcomes and applicable stability.

The advantage of our predictive model is that it is built from a large-population-based data and validated in different countries and regions. Therefore, it can be useful in various clinical settings in different regions. Furthermore, the decision curve analysis showed a greater net benefit for patients that use the clinical prediction model, which can reduce unnecessary wastage of healthcare resources. This is very important today given the emphasis on precision medicine and avoiding over-medication.

In conclusion, we have developed a clinical prediction model to predict overall survival in patients with chondrosarcoma. The clinical prediction model has shown a good predictive accuracy and clinical utility when validated on a dataset consisting of different populations and ethnicities. Thus, this study is the most complete prediction tool for chondrosarcoma designed to date, which can be used by clinicians to predict prognosis, can help stakeholders to screen high-risk patients, can provide valuable reference information for the development of healthcare policies, and can provide assistance for individualized patient counseling, timely monitoring, and follow-up.

## Methods

### Clinical Information and Selection Criteria

Training group data (SEER) were extracted from the SEER database using the SEER*STAT (version 8.3.6) software for demographic characteristics, clinicopathology, and patient treatment (surgery, radiotherapy, and chemotherapy) of patients with incoming chondrosarcoma. The data inclusion criteria were (1) patients with chondrosarcoma who were selected according to the International Classification of Diseases in Oncology, Third Revision histological subtype code: chondrosarcoma, NOS (9220/3); (2) the post-2010 SEER database incorporated information on metastatic sites and therefore included patients diagnosed between 2010 and 2016; (3) chondrosarcoma was the first and only primary malignancy; and (4) complete clinical information, including patient’s age, gender, race, primary site, tumor size, tumor TNM stage and grading, metastatic site, surgery, and whether radiotherapy and chemotherapy were administered.

The validation group data (multicenter data) was from patients with chondrosarcoma who attended four medical institutions from 2010 to 2016, namely, the Second Affiliated Hospital of Jilin University, the Second Affiliated Hospital of Dalian Medical University, Liuzhou People’s Hospital, and Xianyang Central Hospital. These patients were followed up for more than 3 years as the validation group of the prediction model. Three investigators from each institution were responsible for data acquisition during the survey. Tumor size and stage were provided by the surgeon or supervising physician, while pathological grading was diagnosed by a senior pathologist at each hospital. In case of a dispute, the decision was made by the pathologist at the Second Affiliated Hospital of Jilin University. Two of the three investigators extracted the data, and accuracy checks were performed by another investigator.

Complete clinical information as mentioned above was collected from the SEER database, and after screening for inclusion and exclusion criteria, 1,290 osteosarcoma patients between 2010 and 2016 were ultimately included in this study. For the validation data, a total of 104 patients were included in this study after collection and exclusion. The chi-square test and independent samples *t*-test results showed statistically significant differences in the cohort of patients. The ethics committees of all hospitals approved the study. All data were checked for consistency using Microsoft Excel (2016).

### Calibration of Predictive Model Parameters and Data Baseline

This study is a multicenter study, so the cohort of patients from two different countries and different medical centers was standardized as far as possible; the SEER data had three categories for race, namely, white, black, and other—with no specific ethnicity breakdown for other, so the multicenter data from China was also classified as other. The baseline tables were drawn for the training and validation groups, with independent samples *t*-tests for continuous variables and chi-square tests for categorical variables. The heat maps of the data were plotted to show the frequencies and correlations between the parameters.

### Screening of Predictive Model Parameters

Three methods were used to screen the variables in this study: univariate Cox with *P <*0.05 as the cutoff for screening variables for plotting a univariate Cox forest plot; best subset regression to determine the best combination of variables by adjusting for the *R*² maximum; and to find the best combination according to the BSR model evaluation criteria, the Mallows’ Cp minimum is adjusted with R2 maximum and Bayesian information criterion minimum.

LASSO introduces the variable *λ* (lambda, also known as the shrinkage operator, model coefficient ratio, tuning factor, or penalty value) in order to find the best model. The calculation process is presented in the **Supplementary Note**. The LASSO regression is designed to prevent overfitting and to address the problem of severe covariance by generating a penalty function to compress the regression coefficients of the variables in the regression model. Therefore, the *λ* value determines which variables make the model optimal, and cross-validation is used to find the best *λ* value: the *λ* value corresponding to the smallest mean squared error (MSE) determines the variables to be included in the model. The smaller the MSE value, the better the accuracy of the prediction model.

This study is a multicenter study, so the cohort of patients from two different countries and different medical centers was standardized as far as possible. The SEER data had three categories for race, namely, white, black, and other—with no specific ethnicity breakdown for other—so the multicenter data from China was also classified as other. Baseline tables were drawn for the training and validation groups, with independent samples *t*-tests for continuous variables and chi-square tests for categorical variables. Heat maps of the data were plotted to show the frequencies and correlations between the parameters.

The screened variables were included in a multivariate Cox regression using stepwise backward regression to determine the final screened predictors with the minimum AIC to construct the model. The three models were plotted with receiver operating characteristic (ROC) curves at 3 and 5 years as nodes, and the one with the largest AUC was selected as the constructed model.

### Survival Analysis

Kaplan–Meier survival curves were plotted for each predictor of the categorical variables, and log-rank tests were used to determine the significance of differences between the survival curves. Multi-variate Cox regression analysis was used to plot a multivariate Cox forest plot.

### The Development of Predictive Models

A nomogram was constructed using the predictors screened in the model using the results from step 2.3.1. A web calculator was created for ease of use by medical staff or relevant interested parties. A decision tree was also built as a tool to aid the prediction model.

### Validation of the Model and Assessment of Clinical Usefulness

The relationship between the actual and predicted probabilities was verified by plotting calibration curves for the training and validation sets over 3 and 5 years to evaluate the internal and external consistency of the model. The validation set subject ROC curves were plotted and the AUC was calculated to evaluate the prediction accuracy of the prediction model on external data. Risk factor tables and decision curve analysis (DCA) were used to evaluate the clinical application of the column line plots.

### Statistical Analysis

Chi-square test, independent samples *t*-test, LASSO, best subset regression, data heat map, Kaplan–Meier, forest plot, nomogram, web calculator, risk factor association plots, ROC curves, calibration plots, and DCA curves were completed by R, version 4.0.5. Moreover, *p*-values <0.05 were considered to be statistically significant.

## Data Availability Statement

The original contributions presented in the study are included in the article/**Supplementary Material**. Further inquiries can be directed to the corresponding authors.

## Author Contributions

CLY and QC jointly completed the entire research design. WLL,RLW and STD participated in the research and collected and analyzed the data. WLL drafted the manuscripts. HSW and CX provided expert consultation and advice. BW, WYL and ZHH helped polish the language. All authors contributed to the article and approved the submitted version.

## Conflict of Interest

The authors declare that the research was conducted in the absence of any commercial or financial relationships that could be construed as a potential conflict of interest.

## Publisher’s Note

All claims expressed in this article are solely those of the authors and do not necessarily represent those of their affiliated organizations, or those of the publisher, the editors and the reviewers. Any product that may be evaluated in this article, or claim that may be made by its manufacturer, is not guaranteed or endorsed by the publisher.

## Supplementary Material

Supplementary Figure 1 Flow chart of data collection and analysis.

Supplementary Figure 2
**(A)** Correlation heat map between clinical features. **(B)** Heat map about the frequency of clinical features.
