# Supplementary figures and images for: Dynamic Predictive Models With Visualized Machine Learning for Assessing Chondrosarcoma Overall Survival
Source: Front Oncol. 2022 Jul 21;12:880305. doi: 10.3389/fonc.2022.880305 (PMC9351692; doi:10.3389/fonc.2022.880305)

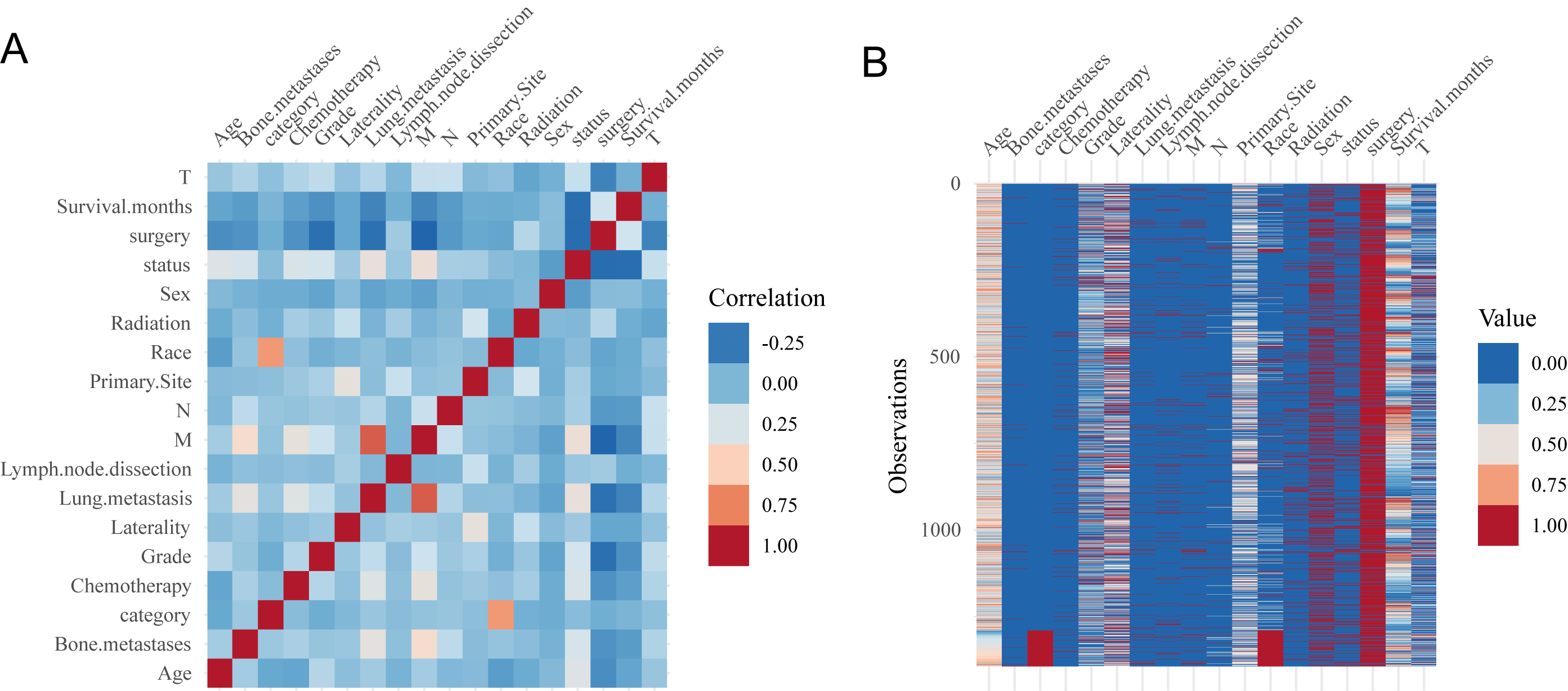

Supplement: Supplementary Figure 2 — (A) Correlation heat map between clinical features. (B) Heat map about the frequency of clinical features. [file Image_2.tif]
